# Critical Points for Interpreting Patients’ Survival Rate Using Cancer Registries: A Literature Review

**DOI:** 10.2188/jea.JE20160180

**Published:** 2018-02-05

**Authors:** Ayako Okuyama, Akiko Shibata, Hiroshi Nishimoto

**Affiliations:** Centre for Cancer Registries, Centre for Cancer Control and Information Services, National Cancer Centre, Tokyo, Japan

**Keywords:** survival analysis, neoplasms, registries

## Abstract

**Background:**

Survival rate is used to develop cancer control plans. However, there are limitations and biases when interpreting patient survival rate data. This study aimed to identify and account for potential biases and/or limitations on estimating survival rate to enable more effective control of cancer.

**Methods:**

The authors searched PubMed from December 2010 to December 2015 for articles that investigated or described biases in estimating patient survival using cancer registries. Articles that only described the tendency of survival rate and investigated relationships between patient characteristics, treatment, and survival rate were excluded.

**Results:**

In total, 50 articles met the inclusion criteria. The identified potential biases were categorized into three areas, as follows: 1) the quality of registry data (eg, the completeness of cancer patients, accuracy of data, and follow-up rates); 2) limitations related to estimated methods of survival rates (eg, misclassification of cause of death for cause-specific survival rate or a lack of comparability of background mortality for relative survival rate); and 3) the comparability of survival rates among different groups (eg, age-adjustment or patients with multiple cancers).

**Conclusion:**

We concluded that survival rate can be suitable for answering questions related to health policy and research. Several factors should be considered when interpreting survival rates estimated using cancer registries.

## INTRODUCTION

Survival rate is used as a measure of cancer burden, and is often employed by policymakers to compare cancer outcomes between different populations and time periods.^1^ Net survival is expected to measure the net effect of a cancer diagnosis after removing the effects of competing causes of death as a cancer prognosis measure.^1^ However, the interpretation of survival rate is not easy. The results of EUROCARE, which is the widest collaborative research project on cancer survival in Europe, showed lower survival rates in the United Kingdom compared with other European countries.^2^ Beral and Peto interpreted trends in mortality from breast cancer as incompatible with the lower survival in the United Kingdom than in other countries.^3^ They hypothesize that the lower reported survival rates in the United Kingdom could arise as an artefact from two main errors: 1) the registration of cancer is not statutory in England and Wales, and a large proportion of cases are registered only because death certificates mentioning cancer are routinely provided to the registries (ie, they infer that when a registration initiated by a death certificate is traced back to obtain clinical data from a hospital, the registry will incorrectly record the data of a recurrence of breast cancer shortly before death, not the correct date of the initial diagnosis); and 2) the United Kingdom survival statistics are falsely low because some long-term survivors are never registered. Woods et al examined how national estimates of survival would change if each of these errors actually occurred.^4^ Their results showed that even implausibly extreme levels of these hypothesized errors in the cancer registry data could not explain the international differences in survival rate observed between the United Kingdom and other countries.

These arguments suggest that we should take into account the potential biases and their effects on estimating survival rate. Also, we should understand the limitations related to the methods used for estimating survival rate when we estimate survival rate using statistical methods (relative survival, and cause specific-survival). This study aimed to identify and account for these potential biases and/or limitations on estimating survival rate, so as to control cancer more effectively.

## MATERIAL AND METHODS

### Data sources

Relevant English language articles published from December 2010 to December 2015 were sourced and extracted using the Medical Subject Headings in PubMed searches. We identified articles using the following combinations of search terms: 1) survival, survival analysis, or survival rate; 2) prognosis and neoplasms; and 3) registries. Manual searches were also conducted for relevant journals (eg, *Journal of Registry Management* and *Journal of the National Cancer Institute Monographs*). Additionally, the referenced articles listed in each of the selected publications were examined.

### Selection of articles

An article was selected only if it fulfilled all of the following criteria: 1) the study estimated or described patient survival using cancer registries (eg, population-based cancer registries or hospital-based cancer registries); 2) the study investigated the potential biases or limitations on estimating patient survival to make international comparisons and to benchmark survival rate, or the study proposed a method to overcome those biases or limitations on estimating survival rate.

An article was excluded if it only focused on describing trends of survival rate in countries or regions. An article was also excluded if it focused on investigating the relationship between patient characteristics, specific treatments, and survival rate.

### Data extraction

We reviewed the titles and abstracts of citations generated from the search to assess their eligibility for further review based on the selection criteria and chose relevant articles for possible inclusion. All selected articles were reviewed and assessed for inclusion in this study. Data was abstracted using a coding sheet that was developed for abstracting relevant parameters, such as registries used, country, methods, and possible biases. All of the authors reviewed the abstracts to ensure completeness and accuracy.

The selected articles were assessed for study quality based on the following points: 1) the aims and objectives of the research were clearly stated; 2) the design of the research was clearly specified and appropriate for the aims and objectives of the research; 3) the researchers provided a clear account of the process by which their findings were reproduced; 4) the researchers displayed enough data to support their interpretations and conclusions; and 5) the method of the analysis was appropriate and adequately executed.^5^

### Data synthesis

The identified potential biases or limitations on estimating survival rate using cancer registries were categorized into three areas: the quality of registry data; the limitations related to estimation methods of survival rates; and the comparability of survival rates among different groups.

All data included in this article were previously published and publicly available. Hence, our study did not require submission to the local institutional review board for ethics approval.

## RESULTS

### Search results and article overview

**Figure 1.**
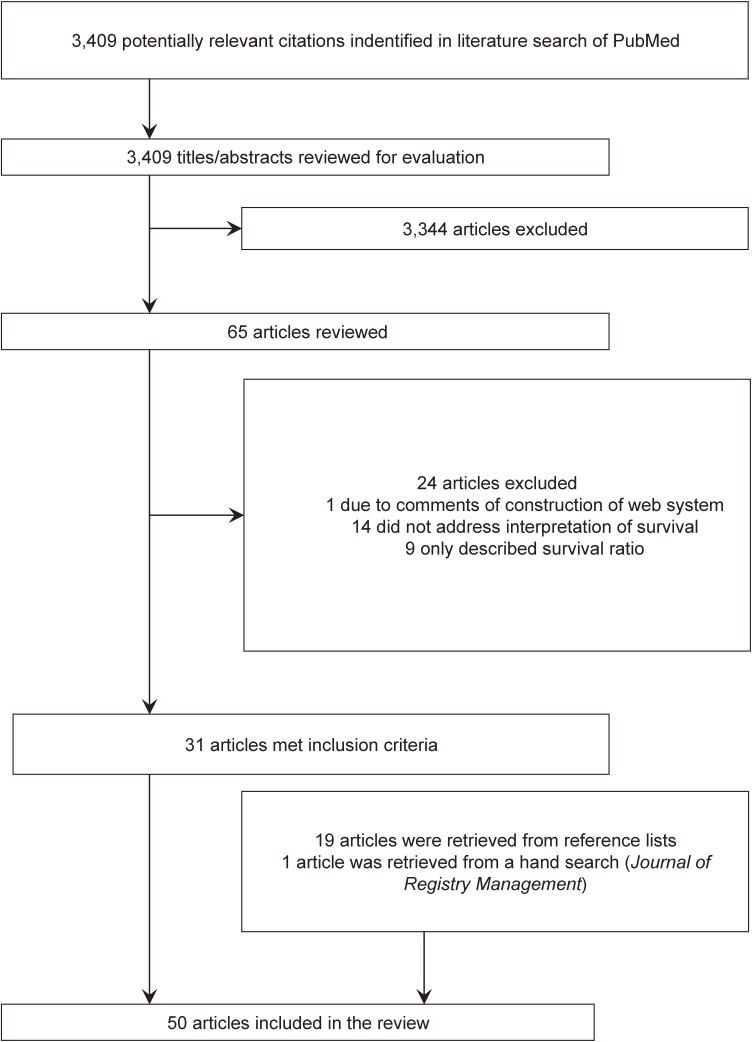
The process of study selection.

Thirty-one articles met our inclusion criteria, and 19 articles were retrieved from the references of selected articles. In total, 50 articles were selected (Figure 1 and eTable 1).^6^^–^^23^^,^^25^^–^^56^ Most studies used data from population-based cancer registries. Four studies used simulated sample data,^19^^,^^25^^,^^26^^,^^55^ and one study used data from a hospital-based cancer registry.^18^

### Quality of registry data

The inclusion of death certificate only (DCO) cases with additional registrations reduced relative survival estimates.^6^^,^^7^ These reductions were more apparent for older patients and for highly fatal cancers.^7^ DCO proportions after successful trace-back of data of diagnosis were positively associated with lung cancer survival rate and negatively associated with colorectal and breast cancer survival rate.^8^ To overcome this potential bias of DCO cases, Silcocks et al proposed a simple method for adjusting the survival estimates with regard to the percentage of DCO cases.^9^ A combination of estimates from both ‘excluded DCO’ and ‘corrected for DCO’ approaches might be useful to delineate a plausibility range for true survival.^10^ The empirical results in Estonia showed that the effect of including/excluding DCO cases from survival analyses was small, except for lung and pancreatic cancers (under the overall DCO rate, approximately 2.0%).^11^ The trace-back can increase inclusion of patients with very poor prognosis, so varying the extent of trace back across registries may compromise comparability of cancer survival rates.^7^ The impact of incompleteness of cancer registration also depends on the history of registries.^12^

The completeness of case ascertainment of cancer registries in the Thames Cancer Registry in England is relatively high, and the impact of case under-ascertainment on estimates of 1-year survival rate were 1.0, 0.8, and 0.4% units for colorectal, lung, and breast cancer, respectively.^13^

Eight articles investigated the impact of incompleteness of vital status information for estimating survival.^14^^–^^21^ The simulation studies showed an impact of loss to follow-up on estimating survival. Brenner and Hakulinen demonstrate that even modest levels of under-registration of deaths may lead to severe overestimation of long-term survival estimates.^16^ The methods of follow-up also have an effect on estimating survival rate.^20^^,^^21^ Survival data from registries using different follow-up procedures are comparable if death ascertainment is complete and all non-deceased patients are presumed to be alive to the end of the study period,^21^ even when the presumed alive method overestimated survival rate compared with the reported alive method by as much as 0.9–6.2%.^20^

The proportion of incomplete registration of death in low- or middle-income countries was relatively higher than that in high-income countries. The loss-adjusted approach, which is estimated under the assumption that the survival of patients lost to follow-up is the same as that for patients with known follow-up time and similar characteristics of different prognostic factors, was proposed.^14^^,^^15^^,^^18^

We also should pay attention to the impact of missing data and imperfect information of the data. Dowing et al showed that, after adjusting for case mix, there were no consistent survival differences amongst the ethnic groups.^22^ Woods et al pointed out that there was a difference in the patterns of excess hazard ratio between the use of partial dates and the use of exact dates for estimating survival rate compared with the value obtained with restricted dates.^23^

Rutherford et al assessed the impact of various cancer registration errors (for example, the initial ‘miss’ of the first true date of diagnosis, and missed follow-up patients) on reported outcomes of cancer survival, and showed differences of up to three percentage units in the 5-year relative survival.^19^

### Limitations related to estimated methods of survival rate (hypothetical setting)

Net survival is not a new concept. A previous study pointed out that net survival is a theoretical measure that can be estimated as either cause-specific survival or relative survival.^24^ Recently, the Pohar-Perme estimator was proposed for estimating relative survival.^25^^,^^26^ This estimator requires no modeling and is accompanied with a straightforward variance estimate.^25^ The Pohar-Perme method does not estimate relative survival. It does estimate net survival in a relative survival setting. Previous methods (eg, Ederer II) estimate survival under the assumption that the excess rate does not depend on the demographic variables. Perme et al pointed out that it was far from being true in most usual situations because the excess hazard was almost always highly associated with age at diagnosis.^25^ The Pohar-Perme method is the only unbiased estimator of net survival. However, this only holds true when the follow-up times are accurately recorded and when there is no informative censoring of the observed survival.^27^ Some researchers regard relative survival (estimated by Ederer II or a modeling approach) as an approach to estimating net survival, because relative survival is biased but the bias in practice is so small that it can be ignored.^27^^,^^28^

The concern for relative survival is caused by a lack of comparability for background mortality between the cancer group and the external general comparison group of the general population. The bias of using expected survival probabilities from the general population will be sufficiently small, so it can be ignored in practical applications.^29^^,^^30^ This bias for common cancer types, older age groups, and all cancers combined was increased up to five percentage units after 10 years of follow-up.^29^ Substantial bias can occur when estimating relative survival rate across subpopulations using non-matching life-tables.^31^ Bakely et al reported that the 5-year relative survival rate using only sex-specific life tables was underestimated by 10–25% for current smoking and Maori populations,^31^ while other researchers reported that using smoking-adjusted life tables to estimate survival has only a small impact on the deprivation gap in survival, even when inequalities in smoking are substantial.^32^^,^^33^ The differences in relative survival for state-specific life tables (SLT) and United States-based life tables (USLT) were generally small, while these differences were slightly higher for states with high social economic status and low mortality and for prostate cancer.^34^

The empirical results show a good agreement between Ederer II and the gold standard (the weighted average of age-specific cumulative relative survival rates, with weights proportional to numbers of patients at diagnosis), while age standardization itself tends to suppress differences that may vary by age.^35^

Cause-specific survival is another hypothetical survival rate. One of the main concerns regarding cause-specific survival estimate is misclassification in cause of death.^36^ Previous studies reported that the differences between cause-specific and relative survival rates for most of the cancer sites was less than 5%, while the differences between cause-specific and relative survival estimates for rare cancer, prostate cancer, good-prognosis cancers, or instances in which the proportion of deaths from other causes was sizeable, were relatively large.^37^^–^^41^

Other studies reported differences in classifications of underlying cause of death, particularly in some subgroups.^42^^,^^43^ For example, Yin et al reported that the patients’ underlying cause of death records disagreed with the California Cancer Registry records in 6% of colon cancer deaths and 39% of rectal cancer deaths, and that after reclassification, the 5-year cause-specific survival rate dropped from 81.2% to 64.9% for rectal cancer.^42^

Period analysis is increasingly used to compute long-term cancer survival, as it provides a better prediction of survival of newly diagnosed patients than traditional cohort analysis.^44^ Period survival estimates may be more prone to bias than cohort estimates when the completeness of the most recent available data is questionable because of delayed recording of some cases.^12^ To describe patient characteristics, all patients who potentially contributed the data to the survival analysis should generally be included in the base of the study.^44^

### Comparability of survival rates among different groups

Age-adjustment of survival rates (hypothetical setting) is employed in international comparisons or in time-series analyses of cancer patient survival, as relative survival rates vary with age for many forms of cancer and the age distribution of cancer patients varies between different populations or within one population over time.^45^ Age-adjusted estimates were less biased in the situation of under-ascertainment.^46^ Standard errors of age-standardized relative survival rate were accurately estimated, while when using Hakulinen’s method, standard errors of non-standardized relative survival rate were overestimated.^47^ Corzziari et al proposed a standard population for comparing survival rates, which consists of three age distributions based on cancer incidence patterns: 1) increasing with age for the vast majority of cancers; 2) broadly constant with age; and 3) mainly affecting young adults.^48^

The different interpretations of the survival rates, both non-standardized and age-standardized, must be known.^49^ Pokhrel and Hakulinen pointed out that “the condition involved is the survival analysis with respect to other causes up to the given point of follow-up. With different periods of follow-up, this condition is also different. As a consequence, the non-standardized relative survival rates and those standardized with the two methods”,^49^^,^^50^ for different periods of follow-up, are mutually incomparable estimates with respect to age.^49^

For the international comparison of cancer survival, complete life tables that are specific for cancer registry area, calendar year, and ethnic background should be used because the background mortality by geographic area, calendar time, ethnicity, age, and sex is quite different.^51^

The proportion of multiple tumors varied greatly by type of tumor, being higher for those with high incidence and long survival.^52^^,^^53^ The inclusion of multiple cancers resulted in lower estimates of 5-year relative survival.^53^^,^^54^ The differences of survival rates between included and excluded multiple cancers cases depends on cancer site and age.^54^

## DISCUSSION

This study demonstrated that several factors should be considered when interpreting survival estimated using cancer registries: the quality of registry data, the limitations related to estimation methods of survival rates, and the comparability of survival rates among different groups.

First, we should assess the quality of registry data. The quality indicators of the data should be available, including information on the percentage of DCO cancers.^20^ The DCO cases occur via two mechanisms: 1) missed diagnosis during the registry coverage period; and 2) cases diagnosed before the start of the registry, which are only found when they die. Recently, researchers pointed out that DCO proportions should be age-adjusted in studies for comparing cancer survival across populations, as the age structure of cancer patient populations has a substantial impact on DCO proportions.^57^ We also took into account the impact of DCO cancers and follow-up rates on estimating survival depending on the cancer site and cancer prognosis. These impacts were increased when we estimated the long-term survival. It can be helpful for policy makers and clinicians to show the results of sensitive analysis, for example, ‘include DCO’ and/or ‘exclude DCO’, as the possible range of the survival estimates. Here, we cannot conclude to what extent the quality of data has an impact on estimating survival, because the background situation in each registry is quite different among countries. However, we should keep in mind that the quality of data has some impact on estimating survival.

There are some arguments for estimating net survival using the Pohar-Perme method.^27^ The Pohar-Perme method is recommended for estimating net survival, as it give unbiased estimates, unlike the traditional relative survival methods.^27^ To estimate 5-year net survival for all ages for a recent period, Dickman and Lambert recommend the Pohar-Perme method because Ederer II applied to all patients may result in a non-negligible bias.^57^ However, the Pohar-Perme method requires follow-up times to be recorded accurately and used as such and when there is no informative censoring of the observed survival. In practice, accurate data of follow-up times are often unavailable and/or there may informative censoring of the observed survival, so we can use Ederer II as well.^27^ In particular, Ederer II can be useful to estimate age-specific survival for a recent period or temporal trends in 5-year survival (age-standardized) within a registry. When we estimate survival rate for older patients or longer-term survival, we should use the Pohar-Perme method with caution. The number at risk as follow-up time increases will reduce proportionately more than other age groups due to higher mortality due to other causes of death and to cancer. In the Pohar-Perme method, these individuals have a lot of weight.^57^

Age adjustment is important for the comparison of survival rates among different groups, as the age distribution of cancer patients varies between the different populations and within one population over time. Corazziari et al proposed the standard population for age-adjustment using the data of EUROCARE.^48^ The distribution of the cancer type could differ between European countries and other countries. Developing a standard population within a country can be helpful for assessing the time trends of the country. In addition, the difference in interpretation between the non-standardized and age-standardized relative survival rates with the strengths and limitations of age-adjustment must be explained to avoid confusion. For example, the population cumulative relative survival curves, consisting of consecutive cumulative relative survival rates, should not be produced for non-standardized rates.^46^^,^^50^

Multiple primary tumors should be included in survival estimates, as the identification of multiple primary cancers can be affected by several factors (eg, the quality and completeness of the surveillance data).^52^^,^^54^ Previous studies showed that survival rate taking into account all primary cancer is more conservative than survival rate taking into account first cancer only.

In this review, most of the included studies used population-based cancer registries. When assessing hospital-based cancer registries for survival estimate, we should keep in mind that those data only cover their sample hospitals.

This study has certain limitations. First, we did not focus on the effect of the change in the coding role, the change in the definition of disease, and/or the stage migration. The survival rate was more sensitive to biases (for example, lead time and length biases) than the population mortality rate. To evaluate the progress against cancer, we must simultaneously interpret trends of incidence, mortality, and survival.^58^^,^^59^ Second, we searched for articles that were published in the last 5 years, so older studies that investigated the potential biases may have been omitted. However, we examined the referenced articles listed in each of the selected publications, so this study could take into account important studies published more than 5 years ago. Finally, half of the selected studies were conducted using cancer registries in Finland and the United States. Therefore, the registries in other countries that have different systems to collect data might show different potential biases.

### Conclusion

Survival rate is a suitable tool to answer questions related to health policy and research. Several factors should be considered for interpreting survival rates estimated using cancer registries. First, we should show the proportion of DCO cancers and followed-up cases. Second, the reasons for using cause-specific or relative survival analysis should be explained. Finally, for the purpose of enabling comparisons between survival rates among different groups, we should undertake age-adjustment and should take into account multiple primary tumors in survival estimates.

## APPENDIX A. SUPPLEMENTARY DATA

eTable 1. Selected articles and quality assessment
