# Supplementary material for: Critical Points for Interpreting Patients’ Survival Rate Using Cancer Registries: A Literature Review
Source: J Epidemiol. 2018 Feb 5;28(2):61–6. doi: 10.2188/jea.JE20160180 (PMC5792228; doi:10.2188/jea.JE20160180)
Supplement: Supplementary file 1 [file je-28-061-s001.pdf]

eTable 1. Selected articles and quality assessment

Author [reference number]	Study aim	Method	Sample and description	Main results	Quality assessment*
**The quality of registry data**					
Brenner H, and Hakulinen T. *Br J Cancer.* 2005:92(3):576-9.[12]	To assess impact of potential bias strongly depends on the time periods affected by under ascertainment and on the type of survival analysis.	To simulate the effects of under ascertainment using scenarios	The Finnish Cancer Registry	The completeness of cancer registry data during various years may be an additional criterion for the choice of either method.	1.Yes 2.Yes 3. Weak 4. Yes 5. Yes
Robinson D, Sankila R, Hakulinen T, Møller H. *Eur J Cancer.* 2007:43(5):909-13.[6]	To assess the impact on survival estimates based on cancer registry data of incomplete ascertainment of cancer cases	To quantify the effects of DCOs and incomplete ascertainment	The Thames (the U.K.) and Finnish Cancer registries (Finland)	The increases in the survival estimates gained from adjusting for incompleteness were for the most part offset by the decrease produced when adjusting for DCOs.	1.Yes 2.Yes 3.Yes 4.Yes 5.Yes
Silcocks P, Thomson CS. *Eur J Cancer.* 2009:45(18):3298-302.[9]	To propose the method for predicting what the likely effect of the trace-back will be on survival and to justify the extra work involved	To demonstrate the model	Trent Cancer Registry in the U.K.	The model (the true survival tends ultimately to (1-p)*S where p is the proportion of DCOs and S is the observed survival. This method works and suggests that researchers should always think about adjusting their survival estimates with regard to the percentage of DCOs.	1.Yes 2.Yes 3.Yes 4.Yes 5.Yes
Brenner H, Holleczek B. *Cancer Epidemiol Biomarkers Prev.* 2011:20(12):2480-6.[10]	To propose alternative" correct for DCO" cases	To develop the model for correcting DCO	Saarland Cancer Registry in Germany	In case of no negligible DCO proportions, cancer survival studies should not exclusively based on either the "exclude DCO" or the "correct for DCO" approach. A combination of estimates for both approaches may be useful to delineate a	1.Yes 2.Yes 3.Yes 4.Yes 5.Yes

				plausibility range for true survival.	
Holleczek B, Brenner H. *Eur J Cancer.* 2012:48(6):797-804.[7]	To assess the impact of trace back on population-based cancer survival estimates	To investigate the survival experience of successfully traced back DCN cases from 1994 to 2003, and assess effect of trace back on population-based 5-year survival estimates	Saarland Cancer Registry in Germany	The inclusion of DCN cancers with additional registrations reduced the 5-year relative survival estimate for all cancers combined by 4% points. Reductions were stronger for older patients and highly fatal cancers.	1.Yes 2.Yes 3.Yes 4.Yes 5.Yes
Lake J, Mark V, Møller H, Davies EA. *Public Health.* 2012:126(1):57-63.[8]	To quantify variation in the estimates across 39 primary care trusts (PCTs) to consider their 1-year cancer survival estimates	1-year relative survival was estimated after direct age standardization using the standard cancer patient population for Europe. Pearson correlation coefficients between survival estimates and death certificate only (DCO) proportions were calculated.	The Thames Cancer Registry between 2002 and 2006 in the U.K.	1-year PCT survival estimates ranged from 6.9 to 19.4 percentage points, and the precision of individual estimates ranged from |0.9| to |6.5| percentage points. DCO proportions were positively associated with lung cancer survival and negatively associated with colorectal and breast cancer survival.	1. Yes 2. Yes 3. Yes 4. Yes 5. Yes
Innos K, Baburin A, Aareleid T. *Cancer Epidemiol.* 2014:38(3):253-8.[11]	To examine recent survival trends in Estonia and to quantify the effect on survival estimates of the temporary disruption	To compare 5-year relative survival calculated from data sets with and without death certificate initiated (DCI) cases	Estonian Cancer Registry in Estonia	The effect of including/excluding DCI cases from survival analysis was small except for lung and pancreatic cancers.	1.Yes 2.Yes 3.Yes 4.Yes 5.Yes

Møller H, Richards S, Hanchett N, et al. *Br J Cancer.* 2011:105(1):170-6.[13]	To assess the impact of incompleteness of the cancer register	To estimate survival using linked routine cancer registration records with information from the Hospital Episode Statistics database	The Thames Cancer Registry (the U.K.)	Completeness of case ascertainment in English cancer registries is 98-99%. The resulting impact on estimates of 1-year survival was small, amounting to 1.0, 0.8, and 0.4 percentage points for colorectal, lung, and breast cancer, respectively.	1. Yes 2.Yes 3. Yes 4. Yes 5. Yes
Sriamporn S, Swaminathan R, Parkin DM, et al. *Br J Cancer.* 2004:91(1):106-10.[14]	To compare loss-adjusted survival probabilities with observed survival	Loss-adjusted survival probabilities were estimated by a logistic regression model with four prognostic factors (age at diagnosis, stage of disease, place of residence and treatment), and compared with observed survival	The population-based cancer registry of Khon Kaen province, Northeast Thailand in 1985-1990	The difference between the loss-adjusted and observed survival at 5 year was small: 2.1% overall, varying between 0.8 and 3.5 per cent units for any prognostic group.	1. Yes 2.Yes 3. Yes 4. Yes 5. Yes
Swaminathan R, Rama R, Shanta V. *Bulletin of the World Health Organization.* 2008:86:509-15.[15]	To measure the bias in absolute cancer survival estimates in the absence of active follow-up of cancer patients in developing countries	Registered incident cases were first matched with those in the all-cause mortality database from the vital statistics division of the Corporation of Chennai. Unmatched incident cancer cases were then actively followed up to determine their survival status.	The population-based cancer registry in Chennai, India during 199-1999 and followed through 2001	Active follow-up of unmatched incident cases revealed that 15% to 43% had died by the end of the follow-up period, while the survival status of 4% to 38% remained unknown. Before active follow-up of cancer patients, 5-year absolute survival was estimated to be between 22% and 47% higher, than when conventional actuarial assumption methods were applied to cases that were lost to follow-up.	1. Yes 2.Yes 3. Yes 4. Yes 5. Yes

Brenner H, Hakulinen T. *Int J Cancer.* 2009:125(2):432-7.[16]	To assess the impact of incomplete registration of deaths through various mechanisms on the validity of long-term absolute and relative survival estimates	To simulate under ascertainment of deaths through linkage failure of registry data with death certificates with probabilities between 0.1 and 5%, and under ascertainment of deaths by unregistered annual emigration with probabilities between 0.05 and 2%	The Finnish Cancer Registry	The results show that even modest levels of under-registration of deaths may lead to severe overestimation of long-term survival estimates, ranging from 0 to 31 per cent units in the scenarios assessed.	1. Yes 2.Yes 3. Yes 4. Yes 5. Yes
Johnson CJ, Weir HK, Yin D, et al. *J Registry Manag.* 2010:37(3):86-103.[17]	To measure the impact of variation in patient follow-up survival statistics	To simulate complete, incomplete, and no follow-up of live patients, and complete and incomplete death ascertainment	The Surveillance, Epidemiology, and End Results Program in US between 1995 and 2000	The 60-month observed survival proportion increased from 54.44% under the original SEER dataset to 54.62% under complete ascertainment of deaths with no follow-up among live patients. Under complete death ascertainment, randomly imputing loss to follow-up among 20% of live cases resulted in a 1% to 2% decrease in 60-month observed survival for 71 of the 102 SEER site categories.	1. Yes 2.Yes 3. Yes 4. Yes 5. Yes
Ganesh B, Swaminathan R, Mathew A, et al. *IARC Scientific Publication.* 2011:162:15-21.[18]	To present formulae that methodologically adjust for losses, and gives examples describing magnitude of bias in survival estimates without such adjustment	Loss-adjusted survival is estimated under the assumption that survival of patients lost to follow-up is the same as that for patients	336 hospital series of treated new breast cancer cases from Mumbai in India	Population-based series comprising 13,371 cases of top ranking cancers from Chennai, with loss to follow-up ranging from 7 to 24%, revealed negligible bias, ranging from 0 to 2% in 5-year survival by the loss-adjusted	1. Yes 2.Yes 3. Yes 4. Yes 5. Yes

		with known follow-up time and similar characteristics of different prognostic factors at first entry		approach for different cancers.	
Weir HK, Johnson CJ, Mariotto AB, et al. J *Natl Cancer Inst Monogr.* 2014:49:198-209.[20]	To assess the effect of different follow-up procedures on five-year survival estimates and develop criteria for identifying high-quality cancer survival data for inclusion in Cancer in North America	To investigate the impact of different follow-up procedures on survival estimates using data from 51 NAACCR member registries with high-quality cancer incidence data.	The Surveillance, Epidemiology, and End Results Program and/or the National Program of Cancer registries in US between 2002 and 2006	The analysis showed that for Canadian and NPCR registries that either conducted national death linkages or met SEER follow-up standards, survival estimates for all races combined appeared to be in the range of the SEER registries.	1. Yes 2.Yes 3. Yes 4. Yes 5. Yes
Pinheiro PS, Morris CR, Liu L, et al. *J Natl Cancer Inst Monogr.* 2014:49:210-7.[21]	To examine biases in survival statistics when comparing the four largest racial-ethnic groups	To compare the "reported alive" method for calculation of survival, which is appropriate when date of last alive contact is available for all cases, with the "presumed alive" method used when dates of last contact are unavailable.	The Surveillance, Epidemiology, and End Results Program in US	The presumed alive method overestimated survival compared with the reported alive method by as much as 0.9-6.2 percentage points depending on the cancer site.	1. Yes 2.Yes 3. Yes 4. Yes 5. Yes

Rutherford MJ, Møller H, Lambert PC. *Br J Cancer.* 2013:108(3):691-8.[19]	To assess the impact of various cancer registration errors on reported outcomes of cancer survival	To simulate to samples of patients diagnosed with cancer from one population and introduce potential registration errors in one of the sample populations under various assumptions	The simulated cohort of size 2,500 over a 5-year diagnosis period	Differences of up to 3 percentage units in the 5-year relative survival proportion (Scenarios, the initial 'miss' of the first true date of diagnosis, linkage error to death register, missed follow up patients, trace-back from death certificates).	1.Yes 2.Yes 3.Yes 4.Yes 5.Yes
Downing A,West RM, Gilthorpe MS, et al. *Ethnicity & Health.* 2011:16(3):201-12.[22]	To investigate the relationship between ethnicity and breast cancer incidence and survival using cancer registry and Hospital Episode Statistics data, and to assess the impact of missing data and the recoding of multiple ethnicities for some patients	To match to multiple episodes, and assess the impact of missing data and the recoding of multiple ethnicities for some female invasive breast cancer patients	The Northern/Yorkshire and West Midlands cancer registry regions in the U.K during the period 1 January 1997-31 December 2003	After adjusting for case mix, there were no consistent survival differences amongst the ethnic groups.	1.Yes 2.Yes 3. Yes 4. Yes 5. Yes
Woods LM, Rachet B, Ellis L, et al. *Int J Cancer.* 2012:131(7):E1120-4.[23]	To investigate bias resulting from the use of partial dates in the estimation of survival	To calculate 1-year survival time for those diagnosed in the same month as their death in two different ways: 1) by assuming that their survival time was 15 days; 2) by excluding zero-month survivors (because their actual survival time is unknown)	The National Cancer Registry for England between 2000 and 2005	There is a striking difference in the patterns of excess hazard ratio is compared to the value obtained with restricted dates (bias of breast 1.1, female colorectal 5.2, male colorectal 3.9, ovary 4.8).	1.Yes 2.Yes 3.Yes 4.Weak 5.Yes

The limitations related to estimated method of net survival

Dickman PW, Auvinen A, Voutilainen ET, et al. *J Epidemiol Community Health.* 1998:52(11):727-34.[41]	To determine the degree to which the choice of survival measure affects the estimation of social class differences in cancer patient survival	Survival rates were calculated by site, sex, and age at 5, 10, and 15 years for each of three measures of survival (relative survival, cause specific survival, relative survival adjusted for social class differences in general mortality)	The Finnish Cancer Registry between 1977 and 1985	The degree of variation in relative survival resulting from social class decreased, although did not disappear, after controlling for social class differences in general mortality. The differences between the three measures were largest when the proportion of deaths from other causes was large, for example, in cancers with high survival, among older patients, and for longer follow up time.	1. Yes 2. Yes 3. Yes 4. Yes 5. Yes
Sarfati D, Blakely T, Pearce N. *Int J Epidemil.* 2010:39(2):598-610.[55]	To compare survival rates using different methods (cause specific survival, relative survival)	To provide simulations relating to the impact of misclassification of death and non-comparability of expected survival for cause specific and relative survival approaches, respectively	Simulated sample	Both cause-specific survival and relative survival are potentially valid epidemiological methods in population-based cancer survival studies, and the choice of method is dependent on the likely magnitude and direction of the biases in the specific analyses to be conducted.	1. Yes 2. Yes 3. Yes 4. Yes 5. Yes
Howlader N, Ries LA, Mariotto AB, et al. *J Natl Cancer Inst.* 2010:102(20):1584-98.[36]	To investigate whether cause-specific survival could be used as an alternative for relative survival, or not	Authors defined cancer-specific deaths according to the following variables: cause of death, only one tumor or the first of multiple tumors, site of the original cancer diagnosis, and comorbidities.	The Surveillance, Epidemiology, and End Results data between 1992 and 2004 in the USA	Authors have developed a classification variable for cause of death associations with specific cancer diagnoses that appears to take into account likely misclassification of cause of death while not overly expanding the causes of death that are associated with each cancer diagnosis. For most of cancer-specific approaches were	1. Yes 2.Yes 3. Yes 4. Yes 5. Yes

		Estimated of relative survival and cause-specific survival that were derived by use of an actuarial method were compared.		similar because life tables were fairly representative of other-cause mortality for most cohorts in this analysis. However, in several situations, one approach provided more reliable results than the others.	
Utada M, Ohno Y, Shimizu S, et al. *Asian Pac J Cancer Prev.* 2012:13(11):5681-5.[39]	To numerically and visually compare survival rates (overall, cause-specific, and relative)	To calculate the proportion of cause of death and 5-year survival rates for lung, liver, or stomach, colon, breast, prostate cancer	The Nagasaki Prefecture Cancer Registry between 1999 and 2003 in Japan	For lung, liver, or advanced stage cancers, the proportions of cancer-related death were high and the differences in survival rates were small. For prostate or early stage cancers, the proportions of death from other causes were high and the differences in survival rates were large.	1. Yes 2.Yes 3. Yes 4. Yes 5. Yes
Hu CY, Xing Y, Cormier JN, et al. *Cancer.* 2013:119(10):1900-7.[37]	To evaluate the utility of the Cause of death in estimating cancer-specific survival and the concordance between relative survival and cause specific survival	The cause of death utility was quantified by using the observed-to-expected (O/E) ratio approach, which was calculated as the SEER-documented observed number of cancer-specific deaths divided by the number of expected deaths attributed to the malignancies as estimated using a relative survival	The Surveillance, Epidemiology, and End Results data between 1988 and 1999 in the USA	In total, 338,445 patients were identified, and their O/E ratios were 0.97, 0.98, 0.90, 1.07, 1.02, and 0.92 for breast, colorectal, lug, melanoma, prostate, and pancreas cancer, respectively. O/E ratios varied slightly with patients' age, race, and tumor stage. The utility of cause of death in calculating cause specific survival depended variously on the risk of cancer-related mortality and non-tumor factors. However, the impact of this variation on cause specific survival was small.	1. Yes 2.Yes 3. Yes 4. Yes 5. Yes

		approach.			
Charvat H, Bossard N, Daubisse L, et al. *Cancer Epidemiol.* 2013:37(6):857-63.[40]	To provide estimates of the crude probabilities of death from cancer and from other causes as well as the probability of being alive up to ten years after cancer diagnosis according to the age and year of diagnosis	To estimate the crude probabilities of death from cancer and from other causes	The data from FRANCIM, the French network of cancer registries	For breast, prostate, lung, and colorectal cancers, the impact of the other causes on the total probability of death increased with the age at diagnosis whereas it remained negligible for lung and head and neck cancers whatever the age.	1. Yes 2. Yes 3. Yes 4. Yes 5. Yes
Skyrud KD, Bray F, Møller B. *Int J Cancer*. 2014:135(1):196-203.[38]	To compare 5-year cause-specific survival estimates and 5-year relative survival estimated for different cancer sites by age and time since diagnosis	Cause specific survival estimates were calculated 1) considering cause of death to be the cancer that was originally diagnosed and 2) considering the cause of death to be a cancer within the same organ system.	The Cancer Registry of Norway between 1996 and 2005	For most cancer sites the difference between cause specific survival and relative survival estimates was small (less than 5%). The greatest differences were seen for rarer cancers such as mediastinum and Kaposi sarcoma.	1. Yes 2.Yes 3. Yes 4. Yes 5. Yes
Yin D, Morris CR, Bates JH, et al. *J Natl Cancer Inst.* 2011:103(14):1130-3.[42]	To examine how often underlying cause of death was misclassified amount colon and rectal cancer patients	Before and after reclassification of misclassified cancer deaths, cause specific survival from colon and rectal cancers was calculated using the life table method.	The California Cancer Registry from 1993 to 1995 in the USA	The patient's underlying cause of death records disagreed with the California Cancer Registry records for 6% of colon cancer deaths and 39% of rectal cancer deaths; 82% of misclassified rectal cancer deaths were misclassified as colon cancer. After reclassification, 5-year cause specific survival for rectal cancer patients dropped from 81.2% to 64.9%.	1. Yes 2.Yes 3. Weak 4. Yes 5. Yes

Schaffar R, Rapiti E, Rachet B, et al. *BMC Cancer.* 2013:13:609.[43]	To describe the difference between the official and revised cause of death variables and the impact on cancer survival estimates	Registrars recoded cause of death based on the rule. The differences between the official and revised cause of death were analyzed, and estimated the impact on cancer survival estimates	The population-based Geneva Cancer registry between 1970 and 2009 in Switzerland	The overall agreement between the official and revised cause of death was high. However, several subgroups presented a lower concordance, suggesting differences in calendar time and less attention given to older patients and more advanced diseases. The impact of discordance on cause-specific survival was small on overall survival but larger for several subgroups (patients with no treatment, patients with hormonal therapy, etc.)	1. Yes 2.Yes 3. Yes 4. Yes 5. Yes
Baili P, Micheli A, De Angelis R, et al. *Tumori.* 2008:94(5):658-68.[51]	To outline how the life tables were constructed for CONCORD; it compares life expectancy at birth between 101 populations covered by cancer registries in 31 countries and compares the impact of two approaches to the development of life tables in relative survival analysis	To study the impact of different approaches, authors compared relative survival in the US using the US national life table, centered on the relevant census years, and the CONCORD approach.	American participating cancer registry for patients diagnosed with breast, colorectal or prostate cancer during 1990-1994	International variation in background mortality by geographic area, calendar time, race, age and sex is wide. Authors suggest that in international comparisons of cancer relative survival, complete life tables that are specific for cancer registry area, calendar year and race should be used.	1. Yes 2. Yes 3. Yes 4. Yes 5. Yes

Talbäck M, Dickman PW. *Eur J Cancer.* 2011:47(17):2626-32.[29]	To calculate expected survival both including and excluding individuals with cancer from the population base, and to estimate the size of the bias arising from using general population estimates	To calculate expected survival both including and excluding individuals with five cancer types	The Swedish Cancer Registry between 1986 and 2002	Authors' evaluation of the bias introduced into the relative survival rates by using expected survival probabilities from the general population shows that for most cancer types the bias will be sufficiently small that it can be ignored in practical applications. This is true when prevalence is low. However, for common cancer types, for older age groups, and for all cancers combined our results show that the bias in the RSR can be up to five per cent units after 10 years of follow-up.	1. Yes 2. Yes 3. Yes 4. Yes 5. Yes
Blakely T, Soeberg M, Carter K, et al. *Int J Cancer.* 2012:131(6):E974-82.[31]	To estimate the bias in relative survival ratios and excess mortality rate ratios from using total population compared to correct subpopulation specific life-tables	5-year relative survival using sex-specific life-table, was compared to the relative survival using fully stratified life-tables (sex, age, ethnicity, smoking status).	The New Zealand Cancer Registration data of 1996-2001 to the 1996 census	5-year relative survival using sex-specific life tables were underestimated by 10-25% for current smoking and Maori populations. Substantial bias can occur when estimating relative cancer survival across subpopulations if non-matching life-tables are used.	1. Yes 2. Yes 3. Yes 4. Yes 5. Yes
Hinchliffe SR, Dickman PW. *Cancer Epidemiol.* 2012:36(2):148-52.[30]	To show how a simple sensitivity analysis can be performed to assess the impact that specific cancer deaths in the population mortality figures can have on the estimate of relative survival	The impact of the specific cancer deaths in the population mortality figures can have on the estimate of relative survival.	The Finnish Cancer registry between 1995 and 2007 and the population mortality data from the Human Mortality Database	The proportion of deaths use to these specific cancers in the general population is so small in comparison to the total mortality that they make little difference to the relative survival estimates. However, prostate cancer proved to be an exception to this.	1. Yes 2. Yes 3. Yes 4. Yes 5. Yes

Hinchiffe SR, Rutherford MJ, Crowther MJ, et al. *Br J Cancer.* 2012: 106(11):1854-9.[32]	To assess the impact that the non-comparability has on the relative survival estimates through the use of a sensitivity analysis	To compare this relative survival with the smoking risk-adjusted relative survival for lung cancer patients	The Finnish Cancer Registry between 1995 and 2007	Although the assumption of comparability between the patient cohort and general population may be unreasonable for lung cancer, authors have shown that correcting for this does not have a concerning impact on the relative survival estimates. In the younger age groups, the probability of dying from other causes is low, therefore, even a fairly large relative adjustment to this value will not have a large impact.	1. Yes 2. Yes 3. Yes 4. Yes 5. Yes
Stroup AM, Cho H, Scoppa SM, et al. *J Natl Cancer Inst Monogr.* 2014:49:218-27.[34]	To assess variations by age, race, and cancer site for all cancers combined, lung, colorectal, prostate, and female breast cancers using state-specific life tables	5-year relative survival was calculated using US-based life tables (USLT) and state-specific life tables(SLT).	17 National Cancer Institute Surveillance, Epidemiology, and End Results Program registries between 2000 and 2009 in the US	Differences in SLT- and USLT-based survival were generally small (less than 4%). Differences were higher for states with high social economic status and low mortality and for prostate cancer. The SLT-based estimates were less reliable than US-based estimates for older populations more than 85 years old.	1. Yes 2. Yes 3. Yes 4. Yes 5. Yes
Ellis L, Coleman MP, Rachet B. *Br J Cancer.* 2014:111(1):195-202.[33]	To describe the methodology for developing a life tables adjusted for smoking, and to assess the impact on net survival estimates and inequalities in survival for laryngeal and lung cancers	Life tables adjusted for smoking were developed, and their impact on relative survival and inequalities in relative survival for laryngeal and lung cancers was examined.	The population-based National Cancer registry of England between 2001 and 2005	Using smoking-adjusted life tables to estimate net survival has only a small impact on the deprivation gap in survival, even when inequalities are substantial.	1. Yes 2. Yes 3. Yes 4. Yes 5. Yes

Jansen L, Hakulinen T, Brenner H. *Br J Cancer.* 2013:108(3):699-707.[44]	To assess which study population should typically be described along with the presentation of period survival estimates by investigating whether the full or restricted cohort has a survival experience that is closer to the period survival estimate	Age-standardized period estimates of 5-, 10-, 15-, and 20-year relative survival were computed for each 2-, 5-,and 10-year calendar period, and compared with survival estimates for two cohorts by means of mean, mean absolute and mean squared differences	Finnish Cancer registry data on 23 common cancer sites between 1954 and 2003	In most computations, survival estimates for the full cohorts were on average closer to the period estimates for the majority of cancer sites. For 10-year survival, results were less obvious with respect to the mean difference.	1. Yes 2. Yes 3. Yes 4. Yes 5. Yes
Hakulinen T, Seppä K, Lambert PC. *Eur J Cancer.* 2011:47(14):2202-10.[35]	To report that the method proposed by Ederer and Heise works well for cumulative relative survival ratios and gives foundation for that finding	To compare the different relative survival methods with the gold standard (the weighted average of age-specific cumulative relative survival ratios with weights proportional to numbers of patients at diagnosis)	The population-based Finnish Cancer Registry between 1970 and 1979	The theoretical and empirical results show a good agreement between the method suggested in 1959 by Ederer and Heise (Ederer II method) and the gold standard. This result is impart due the fact that as follow-up time increases the conditional relative survival ratios become increasingly more independent of age.	1. Yes 2. Yes 3. Yes 4. Yes 5. Yes

Perme MP, Stare J, Estève J. *Biometrics.* 2012:68(11):113-20.[25]	To find the population quantities that the estimators are estimating in the situations when the excess and the population hazard are affected by any common covariates (e.g. age), and to introduce a new estimator of net survival that does not require modeling	To describe population quantities of the traditional estimators and discuss its interpretation, and to propose a new estimator of net survival probability	Simulated sample	It is widely believed that relative survival ratio and net survival represent the same quantity. Whereas this holds when the excess rate does not depend on the demographic variables, it is far from being true in the most usual situation, when this dependence exists. The gap between the two concepts may be large, because the excess hazard is almost always highly associated with age at diagnosis.	1. Yes 2. Yes 3. Yes 4. Yes 5. Yes
Danieli C, Remontet L, Bossard N, et al. *Stat Med.* 2012:31(8):775-86.[26]	To compare estimator abilities to estimate net survival in different settings such as the presence/absence of an age effect on the excess mortality hazard or on the potential time of follow-up, knowing that this covariate has an effect on the general population mortality hazard	To estimate net survival using different methods, and to compare these net survivals	Simulated sample	It showed that when age affected the excess mortality hazard, most estimators, including specific survival, were biased. A multivariable excess hazard model that includes age as covariate and non-parametric which based on the inverse probability weighting take differently into account the informative censoring. These approaches are more suitable for estimating net survival.	1. Yes 2. Yes 3. Yes 4. Yes 5. Yes
Roche L, Danieli C, Belot A, et al. *Int J Cancer.* 2013:132(10):2359-69.[28]	To investigated the magnitude of the errors made by four 'relative survival' methods (Ederer I, Ederer II, Hakulinen, a unavailable regression model) vs. the Pohar-Perme estimator, and to examine the influence of time of follow-up, cancer prognosis, and age on the errors	Net survivals were estimated at 5, 10, and 15 years post diagnosis.	16 participant French cancer registries of FRANCIM network between 1989 and 2004	At 5 years, the errors were generally small. At 10 years, in good-prognosis cancers, the errors made in no standardized estimates with all classical method were generally great (+2.7 to +9% points in prostate cancer) and increased in age-class estimations	1. Yes 2. Yes 3. Yes 4. Yes 5. Yes

	made			(vs. 5-year ones).	
Seppä K, Hakulinen T, Pokhrel A. *Eur J Cancer.* 2015:51(9):1123-9.[27]	To investigate how crucial the assumptions (no informative censoring of the observed survival and use of continuous time) are, when a change of method from the traditional relative to the new net survival is done	A systematic comparison was made against the earlier recommended Eder II method of relative survival using the two currently available computer programs.	The Finnish Cancer Registry between 1981 and 1995	With exact or monthly tabulated data, the Pohar-Perme and the Ederer methods give results that are at five years of follow-up less than 0.5% units and at 10 and 14 years 1-2% units apart from each other.	1. Yes 2. Yes 3. Yes 4. Yes 5. Yes
**The comparability of net survival rate among different groups**					
Brenner H, Hakulinen T. *J Clin Epidemiol*. 2003:56(12):1185-91.[45]	To show derivation of crude and of age-adjusted relative survival rates in the traditional way	To estimate relative survival and to compare these results	The Finnish Cancer Registry	This article illustrates both formally and by hypothetical and empirical examples that derivation and interpretation of crude and adjusted relative survival rates in the traditional way is inconsistent, as it is based on different concepts of what the relative survival rate is intended to measure.	1. Yes 2. Yes 3. Yes 4. Yes 5. Yes
Corazziari I, Quinn M, Capocaccia R. *Eur J Cancer.* 2004:40(15):2307-16.[48]	To define and propose standard cancer patient populations for the age adjustment of cancer survival	A cluster analysis is used for grouping cancer sites according to their similarities in the age distribution of cases.	Over 1.1 million records included in the EUROCARE-2 study	The proposed standard populations consist of three age distributions, appropriate for cancers with incidence patterns: 1) increasing with age, the vast majority of cancers; 2) broadly constant with age; and 3) mainly affecting young adults. The proposed standards were tested on European and US relative survival data. There was very good correspondence between	1. Yes 2. Yes 3. Yes 4. Yes 5. Yes

				the raw and age-standardized survival figures.	
Brenner H, Arndt V, Gefller O, et al. *Eur J Cancer.* 2004:40(15):2317-22.[50]	To propose an alternative approach to age adjustment of both absolute and relative survival rates to overcome both the practical and the conceptual problems inherent in traditional age adjustment	Specific weights are first individually assigned to all patients in different age groups, and one then caries out conventional survival analysis using the "weighted individual data", in which the weighs are applied to the contributions of patients to the numbers of persons at risk and death	The Finnish Cancer Registry	This alternative method overcomes essential shortcoming of the traditional method for age adjustment of relative survival rates. The latter often breaks down if data within single age groups are sparse, in which case it may not be possible to derive age-specific estimates of long-term cancer survival.	1. Yes 2. Yes 3. Yes 4. Yes 5. Yes
Brenner H, Hakulinen T. *Eur J Cancer.* 2005:41(12):1788-93.[46]	To assess the "side-effect" of age adjustment	Various patterns of selective under-ascertainment were simulated, and the bias in crude and age adjusted five-year survival rates were compared.	The Finnish Cancer Registry between 1985 and 1994	Age adjusted estimates were less biased in most scenarios, which may be an additional argument for application of age adjustment in the analysis and reporting of population-based cancer survival rates.	1. Yes 2. Yes 3. Yes 4. Yes 5. Yes

Pokhrel A, Hakulinen T. *Eur J Cancer.* 2009:45(4):642-7.[49]	To investigate the issue (whether the original interpretation of the relative survival ratio is valid for the age-standardized relative survival ratio) for all three proposed methods of age-standardization of the relative survival ratio (Traditional, Benner and Hakulien, Benner and colleagues) *	Non-standardized and age-specific 5-, 10- and 15-year relative survival ratios were calculated for each site and time period for the five groups 0-44, 45-54, 55-64, 65-74, and 75 years and over.	The Finnish Cancer Registry in 1955-1974 and 1985-2004	To avoid over interpretation and confusion, the different interpretations of the relative survival ratios, both non-standardized and age-standardized, must be known. For example, the very popular cumulative relative survival curves, consisting of consecutive cumulative relative survival ratios, should not be produced for the non-standardized ratios or for ratios age-standardized with the two newest methods.	1. Yes 2. Yes 3. Yes 4. Yes 5. Yes
Jansen L, Hakulinen T, Brenner H. *Br J Cancer.* 2012:106(3):569-74.[47]	To assess the validity of the conventional method to estimate standard errors of age-standardized relative survival and to estimate the accuracy of the conventional method when expected survival is computed according to either the Ederer II method or Halulinen's method	Standard errors of mutually comparable non-standardized and age-standardized relative survival were computed by the conventionally used method and compared with bootstrap standard errors.	The Finnish Cancer Registry between 1989 and 1993	When using Hakulinen's method, standard errors of non-standardized relative survival were overestimated by up to 28%. In contrast, standard errors of age-standardized relative survival were accurately estimated. When using the Ederer II method, deviations of the standard errors of non-standardized and age-standardized relative survival were generally small to negligible.	1. Yes 2. Yes 3. Yes 4. Yes 5. Yes
Brenner H, Hakulinen T. *Int J Cancer.* 2007:121(10):2274-8.[54]	To empirically evaluate the dependence of proportion of patients recorded as having a first cancer on time since initiation of cancer registration and the impact of excluding patients with known previous cancer on cancer survival estimates	Authors assessed 5-year relative survival of all patients diagnosed within each calendar period and its change by exclusion of	Finnish Cancer Registry between 1953 and 1997	Among 20 common cancer sites investigated, the proportion of "first cancers" varied between 97.4 and 99.7% in 1953-1957, the first 5-years of cancer registration, and decreased continuously to levels between 83.9 and 92.7% in 1993-1997. Excluding patients	1. Yes 2. Yes 3. Yes 4. Yes 5. Yes

		patients with known previous cancer diagnosis.		with a previous cancer diagnosis had little impact on estimates of survival of cancer diagnosed in 1953-1957, but increased 5-year relative survival estimates among patients diagnosed in 1993-1997 for each of the 20 cancers. The extent of the increase varied by cancer site and age.	
Rosso S, De Angelis R, Ciccolallo L, et al. *Eur J Cancer.* 2009:45(6):1080-94.[52]	To evaluate the impact on the age-standardized relative survival estimates of also including multiple primary tumors	To compare different strategies of analysis:1) first or single tumor: survival indicators were calculated considering the first occurring tumor only; 2) subsequent cancer inclusion: survival indicators were calculated for all tumors whatever their order.	2,919,023 malignant cancers from 69 European cancer registries participating in the EUROCARE-4 collaborative study	The proportion of multiple tumors varied greatly by type of tumor, being higher for those with high incidence and long survival. For all cancers combined the average difference was -0.4 percentage points in women and -0.7 percentage points in men, and was greater for older registries.	1. Yes 2. Yes 3. Yes 4. Yes 5. Yes
Ellison LF. C*ancer Epidemiol.* 2010:34(5):550-5.[53]	To examine the impact of including multiple primary cancers in the derivation of survival estimates	5-year relative survival estimates for persons aged 15-99 years at diagnosis were derived using all eligible primary cases and obtained by using first primary cases	Canadian Cancer Registry from 1992	The inclusion of multiple cancers resulted in lower estimates of 5-year relative survival for virtually all cancers studies. The effect was somewhat attenuated by age-standardization, and was greatest for bladder cancer followed by oral cancer, cancer that had the first and third lowest proportions of first cancers,	1. Yes 2. Yes 3. Yes 4. Yes 5. Yes

		only. Any pre-1992 cancer history of persons on the registry was obtained by using auxiliary information.		respectively.	
Weir HK, Johnson CJ, Thompson TD. Cancer Causes Control, 2013:24(6):1231-42.[56]	To evaluate the effect of Surveillance, Epidemiology, and End Results (SEER) and International Association of Cancer Registries (IACR) MP rules on the population-based cancer survival estimates	To estimate age-standardized relative survival for first cancers-only and all first cancers matching the selection criteria according to SEER and IACR MP rules	Data from 5 US states and six metropolitan area cancer registries participating in the SEER program	This study confirmed the finding from previous studies that survival estimates based on first cancers-only exclude a large, varied, and increasing number of subsequent primary cancers and generally produce less conservative 5-year survival estimates than estimates produced by using all primary cancer coding rules.	1. Yes 2. Yes 3. Yes 4. Yes 5. Yes

CONCORD, global surveillance of cancer survival; DCI, death certificate initiated; DCN, death certificate notification; DCO, death certificate only; EUROCARE, survival of cancer patients in Europe; FRANCIM, The Association of the French Cancer Registries; IACR, International Association of Cancer Registries; NAACCR, North American Association of Central Cancer Registries; NPCR, National Program of Cancer Registries; O/E ratios, observed to expected ratio; PCT, primary care trusts; SEER, the Surveillance, Epidemiology, and End Results; SLT, stage specific life tables; UK, United Kingdom; US, United States; USLT, US-based life tables.

*Quality assessment: 1) Are the aims and objectives of the research clearly stated?; 2) Is the research design clearly specified and appropriate for the aims and objectives of the research?; 3) Do the researchers provide a clear account of the process by which their findings were reproduced?; 4) Do the researchers display enough data to support their interpretations and conclusions?; 5) Is the method of analysis appropriate and adequately explicated?
